# Traumatic bone cyst of the mandible of possible iatrogenic origin: a case report and brief review of the literature

**DOI:** 10.1186/1746-160X-2-40

**Published:** 2006-11-12

**Authors:** Arsinoi A Xanthinaki, Konstantinos I Choupis, Konstantinos Tosios, Vasilios A Pagkalos, Stavros I Papanikolaou

**Affiliations:** 1 Oral Pathology Department, School of Dentistry, University of Athens, Athens, Greece; 2 Oral and Maxillofacial Surgery Department, School of Dentistry, University of Athens, Athens, Greece

## Abstract

The traumatic bone cyst (TBC) is an uncommon nonepithelial lined cavity of the jaws. The lesion is mainly diagnosed in young patients most frequently during the second decade of life. The majority of TBCs are located in the mandibular body between the canine and the third molar. Clinically, the lesion is asymptomatic in the majority of cases and is often accidentally discovered on routine radiological examination usually as an unilocular radiolucent area with a "scalloping effect". The definite diagnosis of traumatic cyst is invariably achieved at surgery. Since material for histologic examination may be scant or non-existent, it is very often difficult for a definite histologic diagnosis to be achieved. We present a well documented radiographically and histopathologically atypical case of TBC involving the ramus of the mandible, which is also of possible iatrogenic origin. The literature is briefly reviewed.

## Background

The traumatic bone cyst (TBC) is an uncommon nonepithelial lined cavity of the jaws. Since it was first described by Lucas[1] in 1929, the lesion has attracted a great deal of interest in the dental literature, but its pathogenesis is still not clearly understood [1-3]. Traumatic bone cysts have been reported in the literature under a variety of names: Solitary bone cyst,[3] haemorrhagic bone cyst,[4] extravasation cyst,[5] progressive bone cavity,[6] simple bone cyst[7] and unicameral bone cyst[8]. The multitude of the names applied to this lesion attests to the lack of understanding of the true aetiology and pathogenesis. The term "traumatic bone cyst" is the most widely used today [2,9,10].

The lesion is mainly diagnosed in young patients most frequently during the second decade of life [4,11-13]. The sex distribution is reported to be quite even [10,11] or men are affected somewhat more frequently [4,12,14]. The majority of TBCs are located in the mandibular body between the canine and the third molar [4,12,14,15]. The second most common site is the mandibular symphysis. Fewer cases are reported in the ramus, condyle and the maxilla, predominantly in the anterior part [11,14,16]. Clinically, the lesion is asymptomatic in the majority of cases and is often accidentally discovered on routine radiological examination [2,4,12,14,17]. Pain is the presenting symptom in 10% to 30% of the patients [4,11,12]. Other, more unusual symptoms include tooth sensitivity[11,13,14], paresthesia[2,18], fistulas[13], delayed eruption of permanent teeth[19], displacement of the inferior dental canal[2] and pathologic fracture of the mandible [20]. Expansion of the cortical plate of the jaw bone is often noted, usually buccally, resulting in intraoral and extraoral swelling and seldom causing deformity of the face. The adjacent to the lesion teeth are usually vital and there is no mobility, displacement or resorption of their roots [2,4,6,11-13]. On radiological examination, a traumatic bone cyst usually appears as an unilocular radiolucent area with an irregular but well defined (or partly well defined) outline, with or without sclerotic lining around the periphery of the lesion. Characteristic for the traumatic bone cyst is the "scalloping effect" when extending between the roots of the teeth. The scalloped outline, however, is often found in edentulous areas also. Occasionally, expansion or erosion of the cortical plate is noted [4,11].

The definite diagnosis of traumatic cyst is invariably achieved at surgery when an empty bone cavity without epithelial lining is observed, leaving very little except normal bone and occasional fibrous tissue curetted from the cavity wall for the histopathologist. Sometimes, the cavity contains a straw-coloured fluid of bright blood [2-4,10,11].

Since material for histologic examination may be scant or non-existent, it is very often difficult for a definite histologic diagnosis to be achieved [2,11,21]. Most of the histologic findings reveal fibrous connective tissue and normal bone. There is never any evidence of an epithelial lining. The lesion may exhibit areas of vascularity, fibrin, erythrocytes and occasional giant cells adjacent to the bone surface [10-12,14,15].

The widely recommended treatment for TBCs is surgical exploration followed by curettage of the bony walls. The surgical exploration serves as both a diagnostic manoeuvre and as definitive therapy by producing bleeding in the cavity. Haemorrhage in the cavity forms a clot which is eventually replaced by bone [4,10-12,14,15,22,23]. It is believed that in some cases there may be a spontaneous resolution [24].

The following is an account of a well documented radiographically and histopathologically atypical case of TBC involving the ramus of the mandible, which is also of possible iatrogenic origin.

## Case report

**Figure 1:**
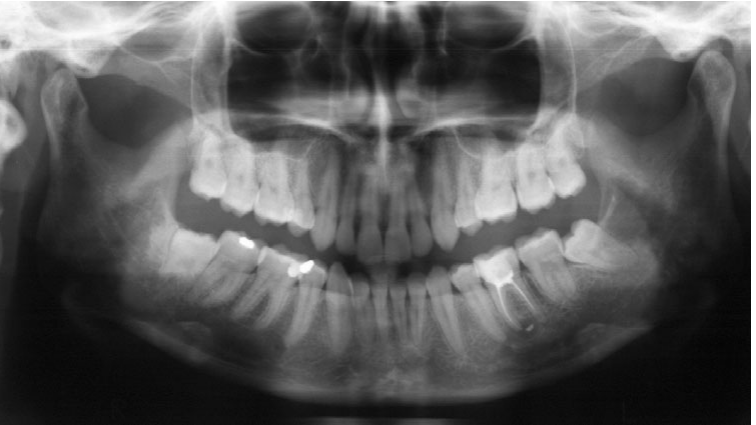
Preoperative panoramic X-ray showing the left lower semi-impacted 3^rd ^molar.

A 25 years white female was referred by her dentist to the Oral Surgery department of the Dental School of Athens University on May 3, 1999, in order to have her semi-impacted lower left 3^rd ^molar surgically extracted. The preoperative panoramic X-ray did not reveal any findings other than the semi-impacted 3^rd ^molar (figure 1). An ID block of the left mandibular nerve together with infiltration anesthesia of the surrounding tissues was given. A triangular mucoperiosteal flap (apex at the disto buccal corner of the second molar) was raised and a periosteal elevator was placed under the periosteum lingually to protect the tissues. Using a surgical bur, adequate bone was removed and the tooth was split and elevated. The wound was thoroughly rinsed with normal saline and sutured using two 3/0 vicryl sutures. No signs of any cystic lesion were noted in the area during surgery. The postoperative recovery was uneventful.

**Figure 2:**
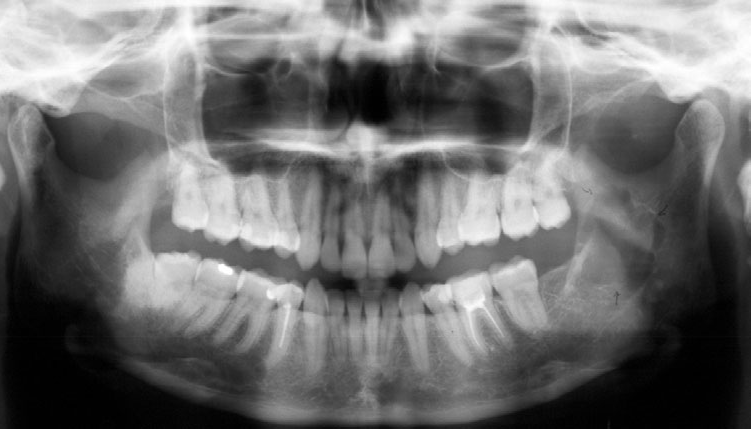
Panoramic X ray taken four years later showing a unilocular radiolucent area in the left ramus.

**Figure 3:**
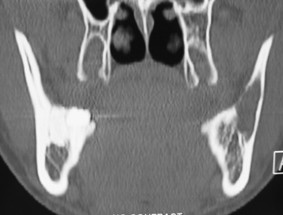
CT scans showed a cyst like low density area in the left ramus region.

**Figure 4:**
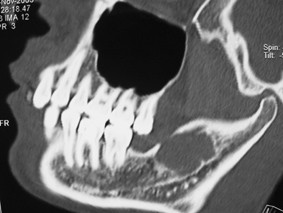
CT scans showed a cyst like low density area in the left ramus region.

Four years later, on October 6, 2003, a routine radiological assessment of the patient with panoramic radiograph revealed a fairly large unilocular radiolucent area in the left ramus sizing 3 × 2.5 cm approximately. The margin of the lesion was slightly irregular. The lesion was partly well defined with radio opaque margin and partly ill defined (figure 2). The patient was completely free of symptoms. There was no expansion of cortical bone, either buccally or lingually. No palpable lymph nodes were present. The medical history was not contributory. A computed tomography (CT scan) showed a cyst-like low-density area in the left ramus region (figures 3 and 4). The differential diagnosis included odontogenic cysts (probably odontogenic keratinocyst) and odontogenic tumors (probably mural or unicystic ameloblastoma).

On November 10, 2003, the patient was operated under local anaesthesia for removal of the cyst. Following an ID block of the left mandibular nerve together with infiltration anesthesia of the surrounding tissues, an incision was made along the external oblique ridge. A mucoperiosteal flap was raised exposing both buccal and lingual surfaces of the ascending ramus. None of the two surfaces presented any noticeable bony expansion. A window was made with a surgical bur in order to reach the lesion. The bony cavity was completely empty of tissue or fluid and there was not any lining on its walls apart from an extremely thin layer of connecting tissue in some places. Following a careful curettage small bone chips with parts of the membrane were submitted for microscopic examination. The operative findings were highly suggestive for the diagnosis of TBC; therefore no further treatment was done apart from curettage.

**Figure 5:**
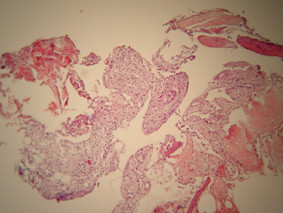
Normal appearing bone spicules with parts of vascular connective tissue (haematoxylin-eosin, original magnification × 40).

**Figure 6:**
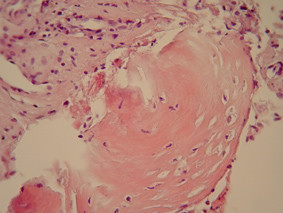
Higher magnification (haematoxylin-eosin × 160).

**Figure 7:**
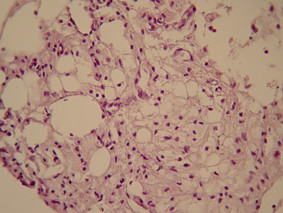
Occasional macrophages were present (haematoxylin-eosin × 160).

Histological examination revealed normal appearing bone spicules with parts of vascular connective tissue (figures 5 and 6). Occasional hemosiderin-laden macrophages were also present (figure 7). The diagnosis was consistent with that of a TBC.

**Figure 8:**
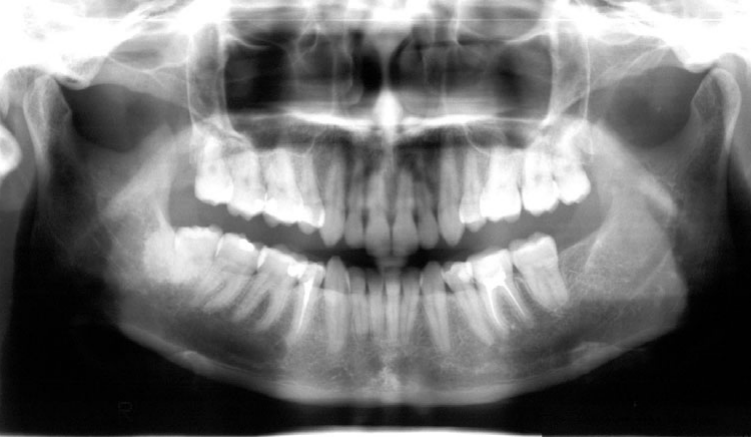
Follow-up panoramic X-ray taken one year after the operation indicates resolution of the lesion.

Postoperative healing was uneventful and follow-up panoramic radiograph on February 16, 2004 indicated restoration of bone structure and resolution of the lesion (figure 8).

## Discussion

In the present case of TBC, the diagnosis is well documented radiographically and histopathologically. It is an interesting case of possible iatrogenic origin which is also located in a rather unusually site, the left ramus of the mandible.

The pathogenesis of the TBC still remains a matter of conjecture and several theories have been suggested. Trauma is the most frequently discussed etiologic factor in the formation of a TBC. Pommer believed that trauma leads to intraosseous hematoma formation. The blood clot liquefies and adjacent bone is destroyed by enzymatic activity [25]. Blum [26] and Thoma [27] believed that a previous traumatic episode to the jaws contributed to the development of most of TBCs. Thoma [27] suggested that trauma initiates a subperiostal hematoma that causes a compromised blood supply to the area, leading to osteoclastic bone resorption.

The traumatic theory that is generally applied to the aetiology of TBC may also be applicable here. In the present case, the surgical extraction of the left lower semi-impacted 3^rd ^molar may have initiated a reaction resulting in the cystic lesion. The extraction of the 3^rd ^molar was performed in 1999, 4 years before the detection of the lesion on routine radiographic examination. The preoperative radiographic examination was negative at that time and no cystic lesion was found in the area during the surgical extraction of the lower left semi-impacted 3^rd ^molar. Thoma [27] stated that a previous definite injury of the affected part of the jaw is contained in the history of most cases and noted that this injury may have occurred several years before the discovery of the lesion. The time interval between the trauma and the discovery of a TBC varies in the literature from 1 week to 20 years [4,11,12]. Howe [4] and Jacobs [28] supported the theory that the content of the cavity depends on the length of time that the cyst has existed. When discovered early, the lesion usually contains blood or serosanguineous fluid. The amount of fluid diminishes with the age of the lesion and eventually becomes empty. In the present case, the cystic lesion was empty. This fact is in agreement with the hypothetic 4 year interval before its discovery.

The presence of a history of trauma is extremely variable in the reported series of cases from 17% [13] to 70% [11]. In Howe's series,[4] over one-half of the patients had a definite history of trauma and the author noted that the severity of the trauma was a striking feature in most of the cases and that this finding suggests that trauma may play a part in the causation of at least a proportion of the TBCs. In some case reports of TBCs, the authors have discussed the possibility of the performed dental extractions to be the responsible trauma factor in their cases. Two of these extractions were considered difficult, whereas none of them was surgical [4,11,26]. In our case, the surgical extraction was not considered to be particularly difficult and the postoperative course was uneventful. We must also note that only few TBCs are seen compared to the number of dental extractions (surgical or not) performed. Blum [27] and Toma [28] believed that there must also be a predisposing idiosyncratic factor in the pathogenesis of TBC, such as a peculiarity of the vessel wall or an abnormal coagulation of the blood [27,28]. Such a predisposing factor could have been involved in our case. Beasley [14] believed that the histological changes observed in their cases of TBCs tended to support the picture of a degenerative process of vascular or neurogenic source of origin and supported the theory that injury or nerve damage within bone results in vascular ischemia and subsequent necrosis to an area. Whether or not such nerve damage occurred during the surgical extraction in our case is unknown.

Another interesting aspect of the present case is its location in the mandibular ramus, one of the least common sites of the lesion. Most TBC cases are located in the body or symphysis of the mandible [2,4,12,15]. Hosseini[29] stated that ''occasionally these lesions may extent into the ramus; however few cases have been reported in a location which is entirely beyond the angle''. Indeed, such atypical lesions, located in the mandibular ramus, condyle or both are rather uncommon in the literature [12,15,29-37]. The atypical location in our case may be due to the involvement of the extraction of the lower left semi-impacted 3^rd ^molar in the pathogenesis of the lesion.

Apart from location, the clinical data in our case are basically in agreement with previous literature. The patient was young (although in the third, not the second decade of life as usually is the case). The lesion was asymptomatic and was discovered accidentally on routine radiographic examination. The radiographic, histopathological and operative findings of the case fit with the literature. Regarding the sex of the patient, some authors tend to disprove the previously reported higher incidence of occurrence in men and believe that sex distribution is quite even [11,10]. Finally, the rapid bone regeneration following the surgical procedure is typical for TBCs [31].

Perhaps the most universal agreement on TBCs is that their aetiology and pathogenesis have not yet been clearly understood. Trauma can be an important factor in the development of TBCs although questions regarding mode, intensity, frequency and pathogenesis must be answered before reaching any final conclusions. Clear, complete and detailed reporting of cases is the only way in which material can be collected for analysis of these problems. In our case, "iatrogenic" trauma appears to be the principal etiologic factor; however, unequivocal proof is lacking.
